# 

**Published:** 1857-09

**Authors:** 


					﻿RECEIPTS ON SUBSCRIPTIONS,
Up to Sept. 10th, 1857.
ILLINOIS.
Dr. L. A. Engle,	vol.	G,	-	$2.00
“ J. B. Meigs,	“	“	-	2.00
44 E. J. Ticknor,	-	44	44	-	2.00
44 A. Holmes, -	44	“	2.00
44 R. L. Goodman,	44	44	-	2.00
“ S. F. Gray,	-	44	-	2.00
“ E. J. French, -	44	“	-	2.00
44 M. C. Archer, -	“	44	-	2.00
“ J. C. Brown, -	“	44	-	2.00
“ J. P. Walker, -	“	“	-	2.00
44 M. V. VanPetten	“	“	-	2.00
44 R. J. Stough	“	“	-	2.00
“ P. A. Allaire, -	“	44	-	2.00
“ P. G. Corkins,	“ 5&G	-	4.00
“ Q. A. Brown, -	44 5&6	-	4.00
INDIANA.
Dr. A. Chapman,	vol. 6,	-	$2.00
“ C.	Badger, -	“	4,5&6,	6.00
“ A.	Woods, -	44	5&6	-	4.00
“ R.	R. Wilson,	- “	6,	-	2.00
Dr. John J. Thompson44 4,5&6,	4.00
44 A. Ber Nebinger,, “	6,	-	2.00
44 J. G. McMakin, 44 4,5 &6,	6.00
44 Porter, -	-	44	6,	-	2.00
IOWA.
Dr. A. B. Ireland, vol. 5,	-	$2.00
44 S. L. Hazen, -	44	44	-	2.00
“J. H. Rice, -	44 5&G -	4.00
44 C. W. Davis,	-	44	44	-	4.00
44 C. H. Rawson, - 44	6,	-	1.00
“ A. McCarty,	-	44	44	-	2.00
44 J. II. Foote,	-	44	4 4	-	1.00
WISCONSIN.
Dr. James Pratt, vol. 5&G,	$3.00
44 R. W. Earle,	-	44	44	-	4.00
44 L. L. Downs,	-	“	6	-	2.00
MICHIGAN.
Dr. F. Knapp, -	vol.	6,	-	$2.00
44 Amos Crosby,	44	“	-	1.00
44 R. S. Haulev,	- 44	44	-	2.00
44 L. W. Lovefl,	44	44	-	2.00
				

